# The correlation between primary open-angle glaucoma (POAG) and gut microbiota: a pilot study towards predictive, preventive, and personalized medicine

**DOI:** 10.1007/s13167-023-00336-2

**Published:** 2023-08-11

**Authors:** Si Chen, Nan Wang, Siqi Xiong, Xiaobo Xia

**Affiliations:** 1 Eye Center of Xiangya Hospital, Central South University, Changsha, 410008 Hunan China; 2 Hunan Key Laboratory of Ophthalmology, Changsha, Hunan China; 3 National Clinical Research Center for Geriatric Disorders, Xiangya Hospital, Central South University, Changsha, Hunan China

**Keywords:** Glaucoma, Gut microbiota, Macrophages, Inflammation, Laboratory medicine, Biomarkers, Predictive preventive personalized medicine (PPPM/3PM)

## Abstract

**Background:**

Glaucoma is the leading cause of irreversible blindness worldwide. Emerged evidence has shown that glaucoma is considered an immune system related disorder. The gut is the largest immune organ in the human body and the gut microbiota (GM) plays an irreversible role in maintaining immune homeostasis. But, how the GM influences glaucoma remains unrevealed. This study aimed at investigating the key molecules/pathways mediating the GM and the glaucoma to provide new biomarkers for future predictive, preventive, and personalized medicine.

**Methods:**

Datasets from the primary open-angle glaucoma (POAG) patients (GSE138125) and datasets for target genes of GM/GM metabolites were downloaded from a public database. For GSE138125, the differentially expressed genes (DEGs) between healthy and POAG samples were identified. And the online Venn diagram tool was used to obtain the DEGs from POAG related to GM. After which GM-related DEGs were analyzed by correlation analysis, pathway enrichment analysis, and protein–protein interaction (PPI) network analysis. Human trabecular meshwork cells were used for validation, and the mRNA level of hub genes was verified by quantitative real-time polymerase chain reaction (RT-qPCR) in the in vitro glaucoma model.

**Results:**

A total of 16 GM-related DEGs in POAG were identified from the above 2 datasets (9 upregulated genes and 7 downregulated genes). Pathway enrichment analysis indicated that these genes are mostly enriched in immune regulation especially macrophages-related pathways. Then 6 hub genes were identified by PPI network analysis and construction of key modules. Finally, RT-qPCR confirmed that the expression of the hub genes in the in vitro glaucoma model was consistent with the results of bioinformatics analysis of the mRNA chip.

**Conclusion:**

This bioinformatic study elucidates NFKB1, IL18, KITLG, TLR9, FKBP2, and HDAC4 as hub genes for POAG and GM regulation. Immune response modulated by macrophages plays an important role in POAG and may be potential targets for future predictive, preventive, and personalized diagnosis and treatment.

**Supplementary Information:**

The online version contains supplementary material available at 10.1007/s13167-023-00336-2.

## Introduction

Glaucoma is the leading cause of irreversible blindness worldwide [1]. It is predicted that there will be over 1.18 hundred million glaucoma patients in 2040 [1]. This disease is characterized as progressive and irreversible loss of retinal ganglion cells (RGCs) mainly due to the increased intraocular pressure (IOP) [2]. The cause of glaucoma is a complex of multiple reasons, such as age and genomic mutations [3–6]. Meanwhile, systemic diseases such as hypertension, thyroid disease, diabetes, and dyslipidemia have been reported to increase the incidence of glaucoma [7, 8]. At present, the clinical methods for early diagnosis and treatment of glaucoma are very limited. Due to the selective retinal ganglion cells (RGCs) damage and characteristic visual field defects, a considerable number of patients with late-stage glaucoma can still maintain a distance vision of more than 5.0, which leads to subjective neglect and missed diagnosis. Meanwhile, in terms of treatment, the intraocular pressure of glaucoma patients is mainly reduced or controlled through 3 methods: medicine, laser, and surgery, to achieve the purpose of protecting the patient’s visual function [9–11]. However, for some glaucoma patients, although the intraocular pressure has been controlled at normal level, visual field defects continue to develop, eventually leading to irreversible blindness. High intraocular pressure is the main factor of glaucoma, but it is not the only factor. The pathogenesis of glaucoma has not been thoroughly studied so far. Moreover, emerged evidence has suggested glaucoma could be considered an immune disorder [12–14]. Glaucoma can be divided into multiple subtypes including primary open-angle glaucoma (POAG), primary angle closure glaucoma (PACG), and secondary glaucoma [15]. Among these subtypes, POAG consist of 74% of glaucoma according to the data from the clinical field [1].In POAG, both the anterior and posterior segments of the eye are affected, and serious damage may be caused upon the trabecular meshwork (TM) and optic nerve (ON) [16]. The trabecular meshwork, located within the iridocorneal angle, is the main pathway for the drainage of the aqueous humor (AH) out of the eye, and its dysfunction leads to IOP elevation [17]. Thus, trabecular meshwork (TM) is the key structure during the process of glaucoma including POAG.

POAG and immune response are not traditionally perceived to be related, but recently, mounting evidence suggests the pathogenesis in POAG is related to immune system disorders [18, 19]. The gut is considered the largest immunological organ in the human body with an irreplaceable role in regulating immune homeostasis [20]. Researches have shown that there is over 10^14^ microbes in the human digestive system: from the stomach to the distal colon, and intestinal microbes are highly varied from individuals [21]. The gut microbiota (GM) and its metabolites have adverse health effects on the human body, and GM dysbiosis can result in a variety of chronic diseases, including metabolic diseases, gastrointestinal diseases, cardiovascular diseases (the gut-heart-axis), and neurodegenerative diseases (the gut-brain-axis) [22–26]. Among them, Flammer syndrome (FS) is a series of special clinical symptoms and signs caused by abnormal blood supply caused by primary vascular dysfunction. Studies have shown that the diseased eating of patients with FS reduces intestinal microflora diversity and negatively impacts the microbiome by evidence of an intestinal dysbiosis, while a dysfunctional GM can further aggravate the symptoms of FS [27].

**Fig. 1:**
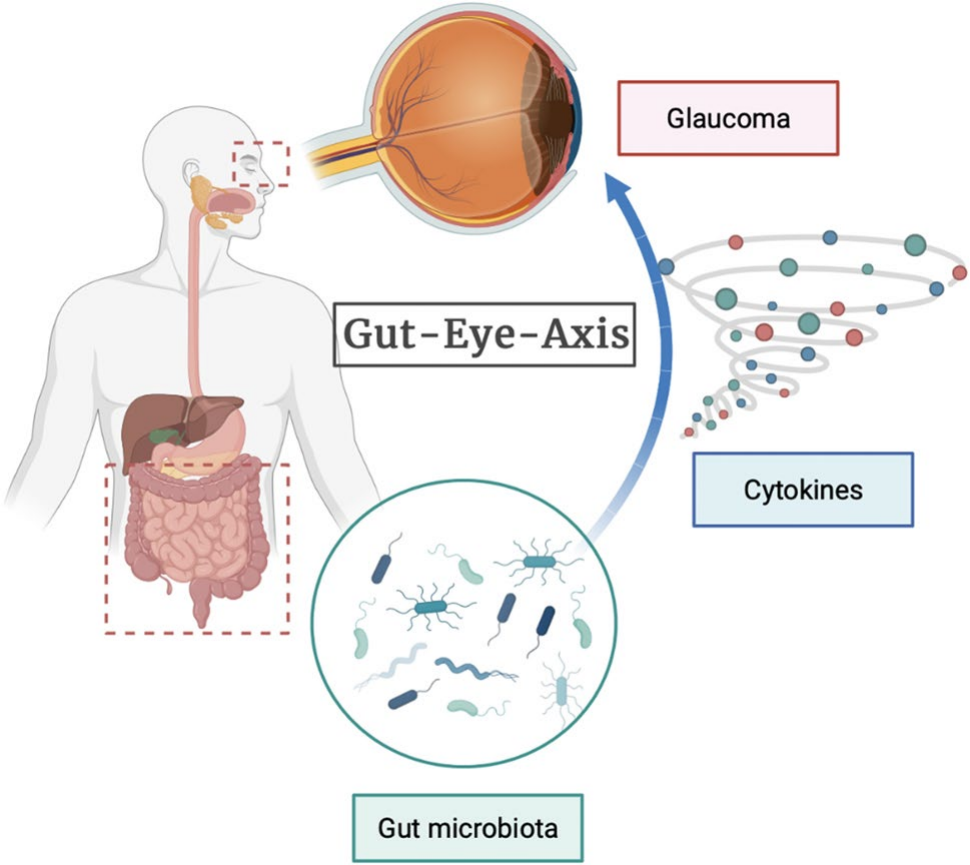
The conception atlas of the gut-eye-axis

Recently, the concept of gut-eye-axis (Fig. 1) have been raised [28], as in human body, biological signals released by the intestinal microbes could lead to immune response from the eye. Researchers have shown series of ocular diseases such as uveitis, macular degeneration [29], diabetic retinopathy [30], and corneal disease [31] relevant to GM dysbiosis. Studies also discovered the possible link between the gut microbiota and glaucoma, for example: irritable bowel disease patients have higher risk of glaucoma [32], and there is distinct difference in gut microbiota composition and serum metabolic phenotype between POAG patients and healthy individuals [33, 34]. Thus, the modification of the gut microbiota in metabolic syndrome and its related chronic diseases stands as a prominent objective within the realm of microbiome research, with the aim of enabling the clinical application of probiotics [35, 36]. The increasing scientific and clinical evidence highlight the impact of microbial influence on distal sites such as the skin, heart, and central nervous system [37]. Despite the above evidence, the knowledge of how the gut microbiota correlated and influenced the process of POAG targeting predictive, prevention, and personalization medicine (PPPM) is still very limited.

## Working hypothesis

Thus, understanding the specific mechanism and mode of action of immune response and immune regulation in POAG will help to obtain a novel approach for the early diagnosis of POAG, to expand effective neuroprotective strategies, and to develop personalized treatment methods, which are in-line with the principle of PPPM. Recently, several studies have revealed how non-ocular factors contribute to the diagnosis of ocular diseases, for example: red blood cell distribution width (RDW) could be a potential laboratory parameter for disease prediction of primary angle-closure glaucoma (PACG) [38]. Moreover, systemic inflammatory biomarkers and metabolomics have been proved to allow early identification and timely implementation of replacement therapies for ocular diseases [39, 40].

In this study, we analyzed RNA-seq data from primary open-angle glaucoma (POAG) patients and co-analyzed them with the genetic data from the gut microbiota database. We found several genes/pathways differentially expressed in POAG patients that are gut microbiota related, among which are critical genes/pathways who played an important role in regulating macrophages differentiation and polarization. Our findings could be regarded as biomarkers for the early identification of POAG and could be developed into a personalized treatment therapy from the prospect of gut microbiota regulation. Our research provided a new perspective to expand the understanding of the gut-eye-axis, the identification of hub genes and pathways could represent potential therapeutic strategies for glaucoma, and point towards future predictive, preventive, and personalized medicine (PPPM/3PM).

## Methods and materials

### Study design and data collection

Datasets of POAG patients were downloaded from the Gene Expression Omnibus (GEO) database (http://www.ncbi.nlm.nih.gov/geo/). The keyword “glaucoma” was used to search related gene expression datasets and non-human tested specimens were excluded. The GSE138125 [41] data set consisted of the gene expression profiles of 4 POAG patients and 4 normal controls. Datasets of the gut microbiota were obtained from gutMGene (http://bio-annotation.cn/gutmgene/home.dhtml), which is a database for target genes of gut microbes and microbial metabolites released by Harbin Medical University.

### Differentially expressed gene analysis

For GSE138125, the differentially expressed genes (DEGs) between healthy and POAG samples were screened by the “limma” package in R software. The genes with *P*-value < 0.05 and logFC > 1 were to be DEGs. In addition, the online Venn diagram tool was used to obtain the gut microbiota-related DEGs (http://bioinformatics.psb.ugent.be/webtools/Venn/). Then, the “heatmap” and “ggplot2” packages in R software are used to draw a heatmap, a volcano plot, and a box plot, respectively, to visualize the gut microbiota-related DEGs [42]. The co-DEGs obtained from the two datasets were visualized using the R packages’ “complex heatmap” and “ggplot2” to generate the heatmaps and volcano maps, respectively. These co-DEGs were retained for subsequent analysis. After screening out the gut microbiota-related genes, the “complex heatmap” and the “ggplot2” R packages were used for visualization, generating heatmaps, and volcano maps, respectively.

### Pathway enrichment analysis and functional annotation

The above differentially expressed gut microbiota-related genes in POAG were submitted to GO function enrichment analysis, which consisted of biological process (BP), cellular component (CC), and molecular function (MF), and the Kyoto Encyclopedia of Genes and Genomes (KEGG) signaling pathway enrichment analysis using an R package “clusterProfiler”. The enriched GO terms and KEGG pathways with an adjusted *P*-value < 0.05 was selected.

### PPI network construction and hub genes identification

The protein–protein interaction (PPI) network was analyzed using the Search Tool for the Retrieval of Interacting Genes (STRING; http://string-db.org). An interaction with a combined score > 0.4 was selected and used to construct a PPI network with Cytoscape software (version 3.9.0). Hub genes were identified for further analysis by using the cytoHubba plugin.

### Cell culture and cell grouping

Human trabecular meshwork cell was purchased from the IMMOCELL company and cultured in DMEM/F12 medium containing 15% fetal bovine serum (FBS) (Gibco, USA) and 1% Antibiotic–Antimycotic (Gibco, USA) at 37 °C with 5% carbon dioxide. When the cell density reached 80%, it was washed with PBS (Gibco, USA) and treated with 0.05% trypsin (Gibco, USA) for passage at the proportion of 1:3.

The logarithmic growth phase cells with good growth condition were inserted into 6-well plates with 1.2 × 10^6^ cells per well. The cells were divided into two groups: the H_2_O_2_-treated group to mimic glaucoma in vitro and the control. The H_2_O_2_-treated group was cultured in the medium containing 200 nM H_2_O_2_, and the normal group was cultured in the SG medium. For H_2_O_2_ treated group, 200 µM H_2_O_2_ was added to each well and cultured at 37 °C for 24 h to create the in vitro glaucoma model.

### RNA extraction and RT-qPCR

RNA was extracted from human trabecular meshwork stem cells using Trizol reagent (Fisher Scientific, #15–596-018) and cDNA generated using the iScript cDNA Synthesis Kit (Bio-Rad Laboratories). For each treatment group, 3 biological replicates were collected. RT-qPCR was performed on a CFX384 Touch Real-Time PCR Detection System (Bio-Rad Laboratories) using the 2X SYBR Green Pro TaqHS Premix (Accurate Biotechnology Co.). Primer sequences are provided in Table 1. Gene expression fold change was calculated using the comparative CT method (2 − ΔΔCT) and the expression in untreated human trabecular meshwork cells was normalized to 1 as the control [43]. GAPDH was used as the housekeeping gene in these experiments.

**Table 1:** Primers sequence

Gene	Sequence
IL18	F ATGCTCTGTTTGGGCTGGATA R GTGAGAGTCGATTTCTGTGGC
NFKB1	F GAAGCACGAATGACAGAGGC R GCTTGGCGGATTAGCTCTTTT
TLR9	F ATGCTCTGTTTGGGCTGGATA R GTGAGAGTCGATTTCTGTGGC
HDAC4	F AGCGTCCGTTGGATGTCAC R CCTTCTCGTGCCACAAGTCT
GAPDH	F GCTTCTCACAAACGAGGACAC R ACTTTCCATGGGTGGAGTCG

### Statistics

RT-qPCR data were analyzed utilizing Prism 9 (version 9.5.0) software, the gene expression fold change was analyzed using a one-way ANOVA and the difference between groups were statistically significant when *P*-value < 0.05.

## Results

In this study, we analyzed RNA-seq data of human trabecular meshwork tissue collected from 4 POAG patients and 4 healthy controls. We further co-analyzed 1606 differential expressed genes (DEGs) screened out from POAG dataset and co-analyzed those DEGs with the gut microbiota database gutMGene v1.0, which contains target genes of gut microbiota and gut microbiota metabolites. The results are presented as follows.

### Identification of GM-related differentially expressed genes in POAG and correlation analysis

**Fig. 2:**
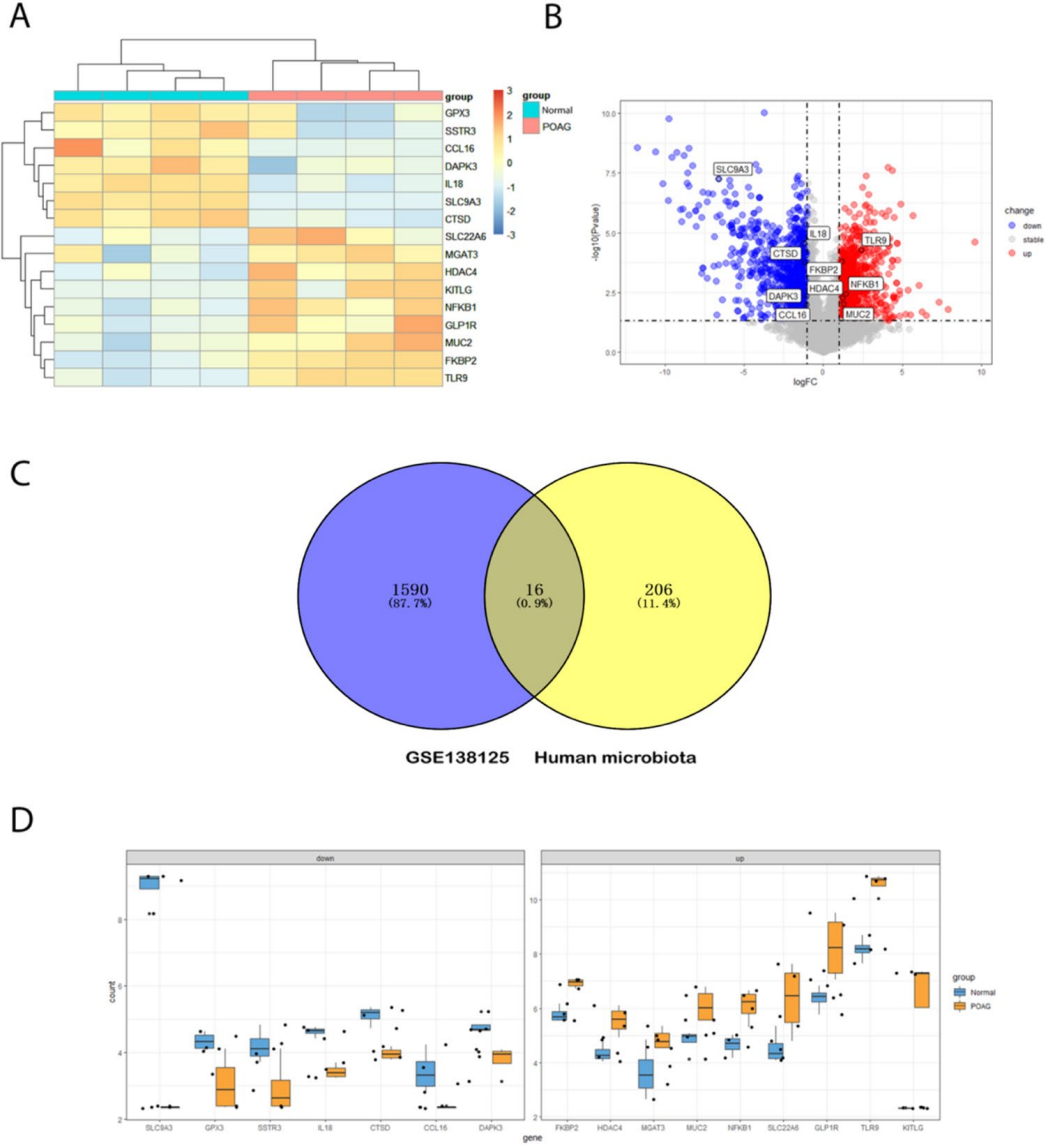
Differentially expressed gut microbiota-related genes in POAG patients and normal control. **A** The heatmap of 16 differentially expressed GM-related genes in POAG patients. Red represents upregulated genes and blue represents downregulated genes. GM, gut microbiota, **B** volcano plot of 16 differentially expressed GM-related genes in POAG patients. The red dots in the picture represent significantly upregulated genes, blue dots represent significantly downregulated genes, black dots represent genes that are not differentially expressed, and the five genes that are most significantly upregulated or downregulated are marked; **C** a total of 201 differentially expressed GM-related genes shown in Venn diagram; **D** the boxplot of 16 differentially expressed GM-related genes in POAG and normal control, including 9 upregulated genes and 7 downregulated genes

1606 DEGs were screened between POAG patients and healthy controls using the “limma” package. Compared with datasets obtained from gutMGene v1.0, a total of 16 differentially expressed GM-related genes in POAG patients were identified after taking the intersection of the Venn, including 9 common upregulated genes and 7 common downregulated genes. These genes were identified as GM-related DEGs in POAG (Fig. 2 and Table 2).

**Table 2:** The 16 differentially expressed gut microbiota (GM)-related genes in POAG samples compared to healthy samples

Gene Symbol	logFC	Changes	*P*-value	Adj. *P*-value	probe_id
TLR9	2.412875125	up	5.08E-05	0.003695358	ASHGV40034404
FKBP2	1.154894525	up	0.000160083	0.007423192	ASHGV40008587
NFKB1	1.453937425	up	0.003557705	0.056479024	ASHGV40039215
HDAC4	1.196327775	up	0.005374753	0.075486516	ASHGV40028293
MUC2	1.299534575	up	0.009370264	0.112888286	ASHGV40007933
KITLG	3.738385125	up	0.015870798	0.162964573	ASHGV40010359
GLP1R	1.889215075	up	0.018638802	0.182172871	ASHGV40045007
SLC22A6	1.80910735	up	0.033283307	0.215021936	ASHGV40008556
MGAT3	1.22276465	up	0.03950615	0.215021936	ASHGV40033182
SLC9A3	− 6.627723	down	5.62E-08	8.17E-05	ASHGV40001585
IL18	− 1.19782	down	2.69E-05	0.002436431	ASHGV40007630
CTSD	− 1.15371	down	0.000174236	0.007880375	ASHGV40006502
DAPK3	− 1.037143175	down	0.004520432	0.066875914	ASHGV40025025
CCL16	− 1.06099675	down	0.009930032	0.117390597	ASHGV40020203
GPX3	− 1.273918675	down	0.020037633	0.190879883	ASHGV40042571
SSTR3	− 1.2638477	down	0.02652658	0.215021936	ASHGV40056858

**Fig. 3:**
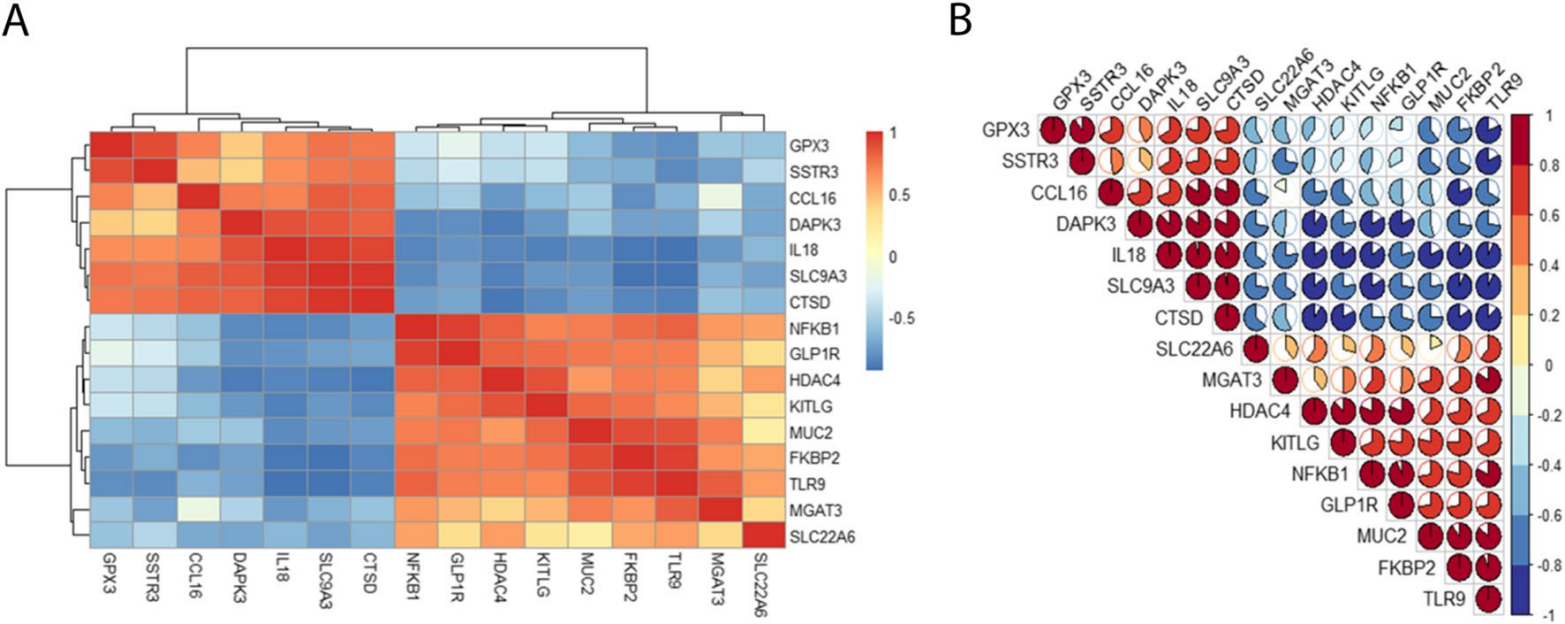
Correlation analysis of 16 differentially expressed GM-related genes in POAG patients and normal control. **A** and **B** correlation heatmap

To explore the expression correlation of these 16 GM related DEGs in POAG, the correlation analysis has been performed by bioinformatics methods. The results showed that there was a high correlation between upregulated genes and downregulated genes, respectively (Fig. 3).

### Pathway enrichment analysis

To further explore the underlying biological processes of these GM related DEGs in POAG, gene ontology (GO) enrichment analysis, Kyoto Encyclopedia of Genes, and Genomes (KEGG) pathway enrichment analysis were performed. The results showed that in terms of GO analysis, the genes were mainly enriched in signaling pathways related to regulation of macrophage activity such as: positive regulation of granulocyte macrophage colony stimulating factor production, granulocyte macrophage colony stimulating factor production, positive regulation of macrophage-derived foam cell differentiation.

**Fig. 4:**
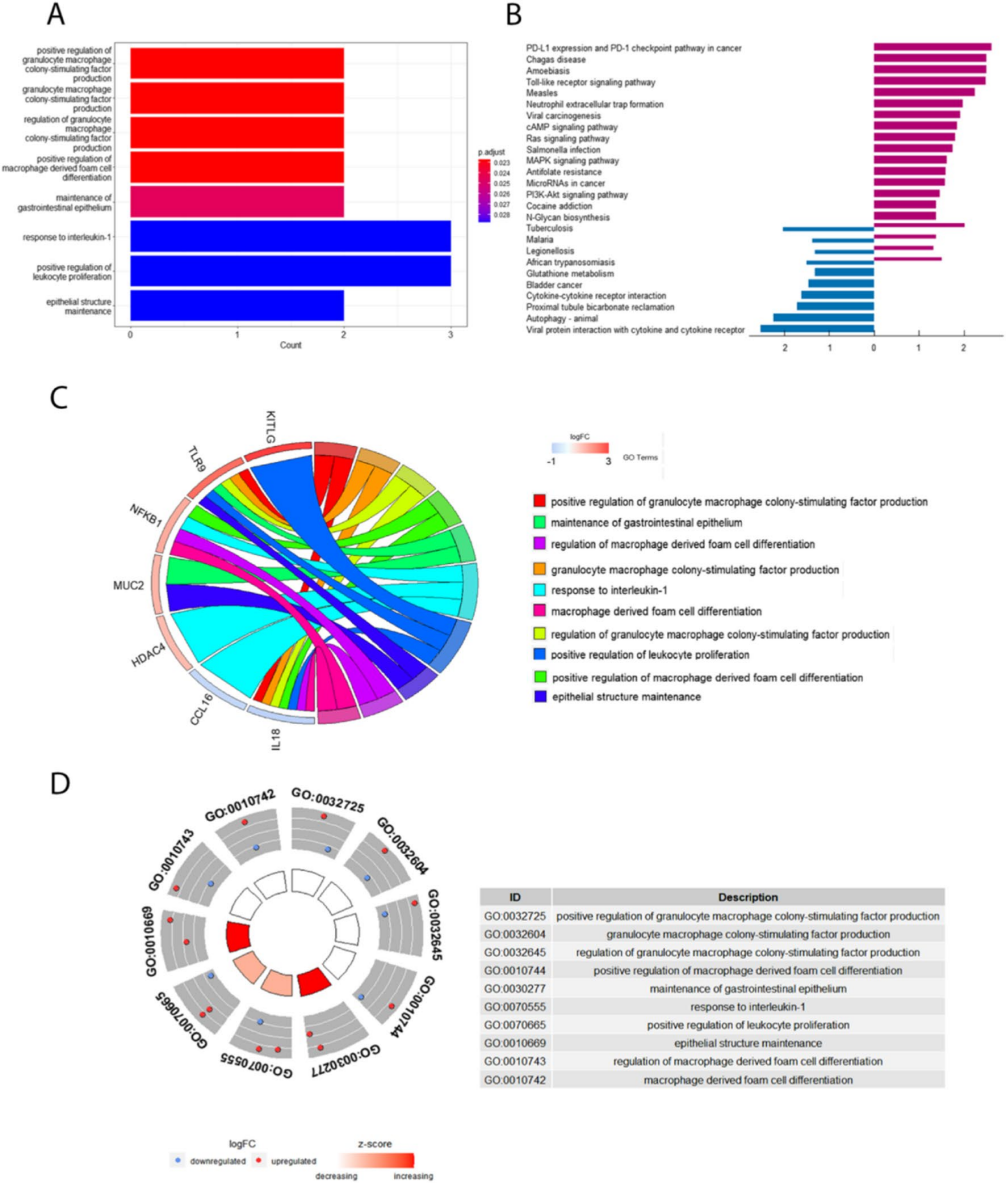
Pathway enrichment analysis of 16 differentially expressed GM-related genes in POAG, including BPs, CCs, and MFs. **A** Bar plot of the enriched GO terms, **B** bar plot of enriched KEGG terms, and **C** chordal graph of the enriched GO terms. It shows the correlation between the upregulated differentially expressed GM-related genes in POAG and the first 10 enriched GO pathways. **D** Eight diagrams of the enriched GO terms. GO, gene ontology; BPs, biological processes; CCs, cellular components; MFs, molecular functions

Meanwhile, KEGG analysis showed that DEGs related to GM were mainly enriched in PD-L1 expression and PD-L1 checkpoint pathway in cancer and a series of parasite diseases including Chagas disease and Amoebiasis (Fig. 4 and Table 3).

**Table 3:** Functional and pathway enrichment analyses for differentially expressed gut microbiota (GM)-related genes in POAG

Term	Description	Count	*P*-value	Adj. *P*-value	Genes
Biological processes					
GO:0032725	positive regulation of granulocyte macrophage colony-stimulating factor production	2	7.14E-05	0.022724817	TLR9、IL18
GO:0032604	granulocyte macrophage colony-stimulating factor production	2	9.24E-05	0.022724817	TLR9、IL18
GO:0032645	regulation of granulocyte macrophage colony-stimulating factor production	2	9.24E-05	0.022724817	TLR9、IL18
GO:0010744	positive regulation of macrophage derived foam cell differentiation	2	0.000116091	0.022724817	NFKB1、IL18
GO:0070555	response to interleukin-1	3	0.000227134	0.028746332	NFKB1、HDAC4、CCL16
GO:0070665	positive regulation of leukocyte proliferation	3	0.000261452	0.028746332	TLR9、KITLG、IL18
Cellular component					
GO:0035580	specific granule lumen	2	0.001153971	0.044166957	NFKB1、CTSD
GO:0045121	membrane raft	3	0.002366087	0.044166957	SLC22A6、CTSD、DAPK3
GO:0098857	membrane microdomain	3	0.002366087	0.044166957	SLC22A6、CTSD、DAPK3
Molecular functions					
GO:0005126	cytokine receptor binding	4	7.33E-05	0.007407743	TLR9、KITLG、IL18、CCL16
KEGG pathway					
hsa04623	Cytosolic DNA-sensing pathway	2	0.005025394	0.092589797	NFKB1、IL18
hsa05321	Inflammatory bowel disease	2	0.005341719	0.092589797	NFKB1、IL18
hsa05235	PD-L1 expression and PD-1 checkpoint pathway in cancer	2	0.009823429	0.114747773	TLR9、NFKB1
hsa04061	Viral protein interaction with cytokine and cytokine receptor	2	0.012284424	0.114747773	IL18、CCL16
hsa04620	Toll-like receptor signaling pathway	2	0.013240128	0.114747773	TLR9、NFKB1

### Relationship between enriched pathways

**Fig. 5:**
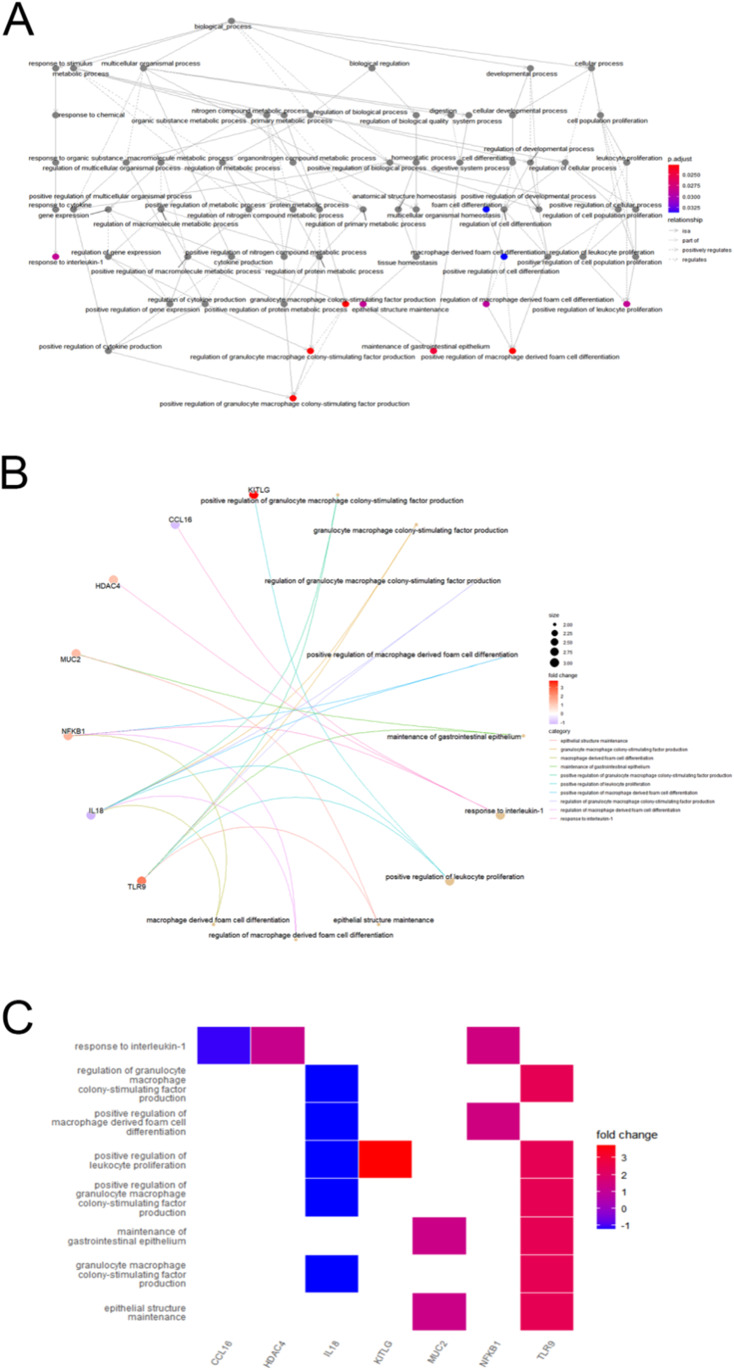
Correlations between the enriched pathways **A** relationships between the enriched GO pathways, **B** common genes in the top enriched GO pathways, **C** heatmap–like functional classification. Genes that are most significantly upregulated or downregulated are marked

The relationship between the above enriched pathways was shown (Fig. 4). There were 7 common GM-related genes in POAG patients in the 10 most prominent pathways, which are CCL16, nuclear factor kappa B subunit 1(NFKB1), interleukin 18(IL18), KITLG, TLR9, MUC2, and HDAC4. The 10 most prominent pathways include positive regulation of granulocyte macrophage colony-stimulating factor production, maintenance of the gastrointestinal epithelium, regulation of macrophage-derived foam cell differentiation, response to interleukin 1, and positive regulation of leukocyte proliferation. Besides, we analyzed the expression of differential genes in the significantly enriched pathway and showed the results in the heatmap-like functional classification map (Fig. 5).

### PPI network construction of GM related DEGs and identification of hub genes

**Fig. 6:**
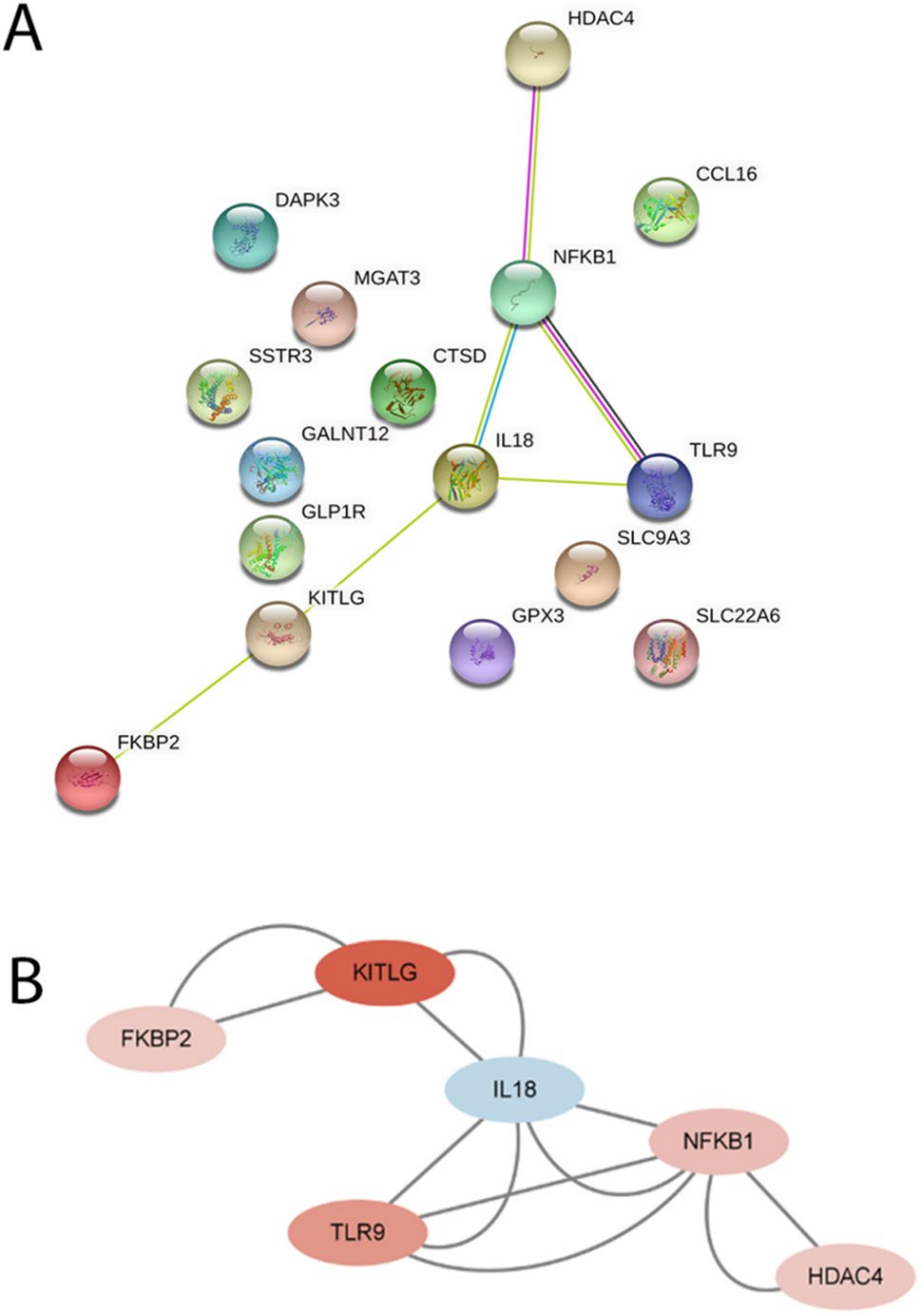
PPI network and identification of hub genes. **A** PPI network of the differentially expressed GM-related genes in POAG patients, constructed by STRING. **B** Significant gene module. STRING, Search Tool for the Retrieval of Interacting Genes

To discover the potential relationships between proteins encoded by GM-related DEGs in glaucoma and to identify hub genes, the PPI network of the DEGs was screened by Search Tool for the Retrieval of Interacting Genes (STRING), including 16 nodes and 6 edges, with a PPI enrichment *P*-value < 0.0153 (Fig. 6A). Then, the obtained results were imported into Cytoscape software for visual analysis. The interaction number of each gene was also shown. The PPI network was analyzed by Cytoscape plug-in CytoHubba to identify hub genes (Fig. 6B). The top 6 genes identified as potential hub genes are: nuclear factor kappa B subunit 1(NFKB1), interleukin 18(IL18), KITLG, TLR9, FKBP2, and HDAC4.

### Validation of the hub gene expression in the in vitro glaucoma model

**Fig. 7:**
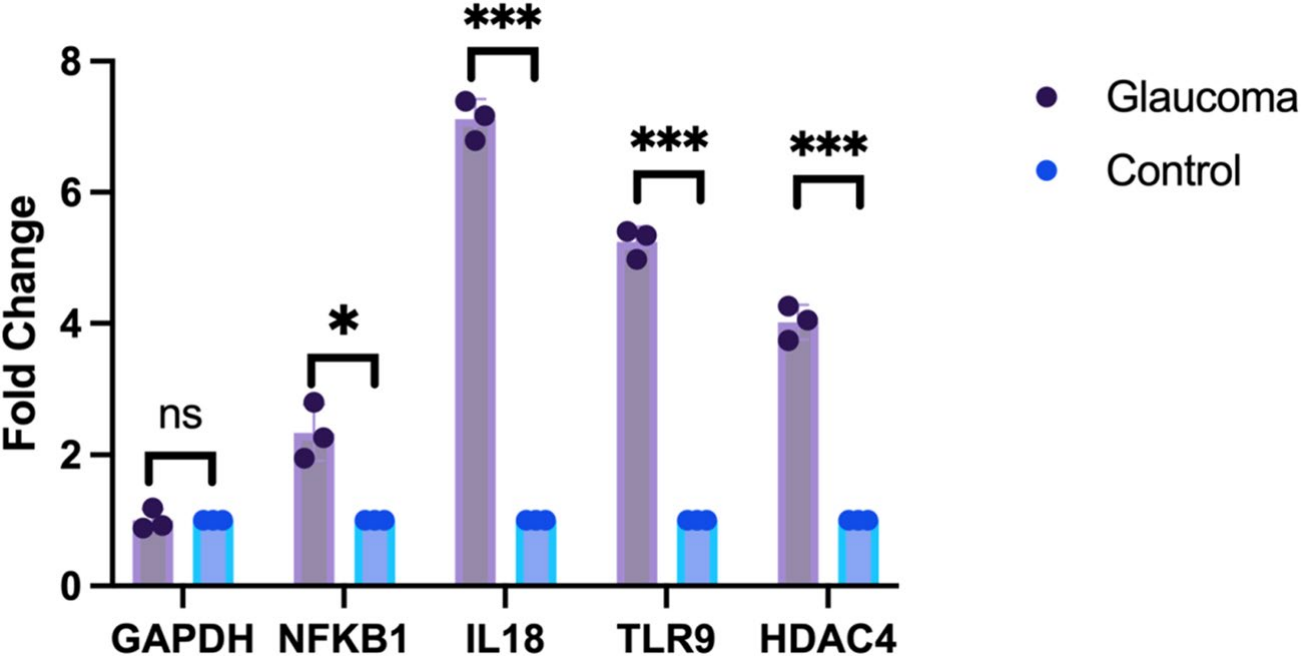
The mRNA level of 4 hub genes. The mRNA level of NFKB1, IL18, TLR9, and HDAC4 were measured in human TM cells by RT-qPCR. *P*-values were calculated using one way ANOVA. *P < 0.05; ***P < 0.001; ns, non-significant; RT-qPCR, quantitative real-time polymerase chain reaction

In this study, we incubated human trabecular meshwork cells with H_2_O_2_ (200 µM) to simulate a glaucoma model in vitro. We performed RT-qPCR to validate the expression change of the hub genes IL18, HDAC4, TLR9, and NFKB1. NFKB1, TLR9, and HDAC4 were predicted to be upregulated in glaucoma and their mRNA level increased according to the RT-qPCR data (Fig. 7), meanwhile, IL18 was predicted to be downregulated in glaucoma and its mRNA level was also increased according to RT-qPCR.

## Discussion

Glaucoma has a huge impact on public health worldwide and primary open-angle glaucoma (POAG) is responsible for over 70% of glaucoma [8]. Current consensus on glaucoma is not solely based on intraocular pressure (IOP), especially in cases of normal-tension glaucoma (NTG). Although high IOP is a well-established risk factor for glaucoma, there is increasing evidence that other factors, such as vascular and neurodegenerative mechanisms [19, 20], also play a role in the development and progression of the disease. Lowering IOP is currently the only intervention available [21]. However, clinical evidence indicates that lowering IOP does not prevent progression in all POAG patients [22]. Thus, non-IOP factors are involved in POAG. Considering these findings, the treatment of glaucoma has shifted from a sole focus on lowering IOP to a more individualized approach that considers other factors that may be contributing to the disease. Previous studies have revealed inflammation and immune response mediate the process of POAG from several different aspects [9], yet much remains to be understood with regards to cellular processes regulating the maintenance of the TM and its relevance to IOP.

Recent advances have identified the significant associations between the gut microbiota and several disorders in multiple organs besides the digestive tract [44, 45]. Emerging evidence has suggested the gut microbiota could be the regulator of several central nervous system disorders, including the ocular system disorders [46]. For example, the gut microbiota has been recognized as an important contributor to anorexia nervosa as an extreme cases of the FS phenotype, furthermore, FS is strongly associated with glaucomatous optic neuropathy [27]. Although data from the clinical field are showing the possible link between the gut microbiota and the occurrence of POAG [33, 47–49], the mechanism underlying its connections and the key molecules remained unknown.

In this study, we aimed at discovering the key genes and pathways in POAG that are gut microbiota related. After analysis, NFKB1, IL18, TLR9, FKBP2, KITLG, and HDAC4 were recognized as hub genes in POAG patients and in the database of gut microbiota regulation. They showed the most remarkable correlation with regulation and stimulation of leukocytes such as macrophages. Thus, we conclude these 6 hub genes might play important roles via affecting the activity of macrophages during the progression of POAG and in regulating gut microbiota. We further verified the expression level of 4 hub genes (NFKB1, TLR9, HDAC4, and IL18) through RT-qPCR. The above 4 hub genes were shown to be regulating immune response from different aspects. Based on our RT-qPCR results, the mRNA level of all 4 genes was increased in H_2_O_2_-treated group compared to the control.

NFKB1 has been reported to be playing a critical role in suppressing inflammation, aging and cancer [50]. NFKB1^−/−^ mice have reduced numbers of conventional and plasmacytoid dendritic cells in response to TLR9 stimulation [51]. Moreover, NFKB1^−/−^ bone marrow-derived macrophages show an increased expression of interferon-inducible genes, including IFN-β, in response to TLR-4 and TLR-9 stimulation [52]. Toll-like receptor 9 (TLR9) is an intracellular TLR, expressed in different immunological and non-immunological cells [53]. The gut microbiota is a source of TLR ligands, including TLR9. Though its role in glaucoma via regulating gut microbiota remained mystical, previous research has indicated that TLR9 deficiency induces osteoclastic bone loss via gut microbiota-associated systemic chronic inflammation [54], thus, it is possible that the TLR9 mediate the occurrence of glaucoma through chronic inflammation process. Targeting immune responses mediated by TLR9 is a potential therapeutic strategy for preventing disease-associated inflammation and autoimmune diseases [55]. Several previous studies have also described a wide range of oxidative stress-related makers which are found in glaucomatous patients, including the activation of the NFKB pathway and the upregulation of pro-inflammatory cytokines [56].

Little is known of what the function of HDAC4 in glaucoma is, but emerging evidence has shown HDAC4 has its importance in regulating neurogenesis [57]. In vivo selective inhibition of HDAC4 via MS-275 (entinostat) or LMK-235 could prevent ongoing RGC degeneration [58]. Reduced HDAC4 expression is associated with blood–brain barrier (BBB) breakdown contributing to ischemia/reperfusion injury-induced infarct in ischemic stroke model rats, while increased HDAC4 expression ameliorates BBB injury, contributing to the reduced infarct volume [59].

IL18 is a pro-inflammatory cytokine which has varies function in different cell types [60]. As an important regulator for both innate and acquired immune response, IL18 plays an important role in inflammatory/autoimmunity diseases. Research has shown the intestinal epithelial cell secretion of IL18 is necessary for gut microbiota homeostasis (dysbiosis) [61]. Meanwhile, increased IL18 expression was observed in glaucomatous trabecular meshwork [62]. Our bioinformatic prediction showed downregulation of IL18 while the RT-qPCR result showed significantly increased mRNA level of IL18, and RT-qPCR result is consistent with the most previous research. In that case, we hypothesized that the reason for the downregulation of IL18 showed in bulk RNA-seq data is due to the small sample number.

Pathways enriched in both GO and KEGG database are mostly related to the activity of leukocytes especially macrophages. Substantial amount of research concerning the role of the immune system in glaucoma has been performed in the recent years. Researchers have provided evidence of macrophages participating in the process of glaucomatous pathogenesis [63], studies analyzing the trabecular meshwork of patients with POAG found macrophages in this tissue by scanning electron microscopy [64], and the presence of macrophage inhibitory factor were observed in healthy human doners’ TM. Meanwhile, it is shown that using macrophage-activator drug Zymosan could induce neurodegenerative effect in glaucoma model [65]. Moreover, a study has revealed enhanced expression of vascular regulation gene ABC1 in leukocytes as a potential marker for the diagnostics and ex vivo molecular monitoring of glaucoma [66]. Nevertheless, TM has been shown to display characters of several cell types including expressed behavior pattern of macrophages when it operates to maintain homeostasis of IOP [67], which further support the accuracy of our bioinformatic analysis. It seems like macrophages played the role of the “double sided sword” in glaucoma. Still, deeper discovery of the protective factors of the macrophages is needed to bring forward a therapeutic approach.

In this study, we utilized a hydrogen peroxide (H_2_O_2_) model to mimic glaucomatous ocular hypertension in vitro. H_2_O_2_ induces acute oxidative stress to TM which leads to the pathogenesis of POAG [68], this model was proved to be a useful platform which resulted in TM cellular dysfunction without the destruction of the TM structure [69], which made this model suitable for the hypothesis that gut microbiota dysbiosis regulates TM cellularity through innate immune response.

Gut microbiota and its metabolites contribute to a variety of diseases with inflammation components via the regulation of macrophages, for example, intracranial aneurysm [70], IBD (inflammatory bowel disease) [71], Alzheimer’s disease [72], and more. Gut microbiota-derived metabolite SCFAs (short-chain fatty acids) promotes anti-inflammatory functions of the macrophages [73], as another gut microbiota metabolite TMAO (trimethylamine-N-oxide) induces M1 macrophage polarization [74]. Specific strains such as *L. delbrueckii* subsp. *bulgaricus* IMV B-7281 and *Lactobacillus* spp have been shown to significantly stimulate macrophages to synthesize inflammatory factors such as IL-12, which plays a key role in the activation of cell-mediated immunity. Besides, the heterogeneity of the bacterial cell wall of these probiotics can be used as an ideal biomarker for predicting host-bacterial interaction, which has important clinical significance to facilitate the development of personalized probiotics and probiotics treatments [74]. Our research showed that the 6 most critical glaucoma pathogenic genes, such as the NFKB1, IL18, and TLR9, are closely relevant to strains such as *Bifidobacterium adolescentis* and *Lactobacillus paracasei* JS1, which may affect the progression of the disease through metabolites such as Equol, Butyrate, and Indole (Supplement Fig. 1). Currently, it is still unclear how gut microbiota effects glaucoma via the regulation of macrophages, it will be crucial to testify this mechanism in the real world. The gut-eye-axis is a relatively new concept that has gained attention in the scientific community in recent years.

Utilizing non-ocular laboratory examinations to help with early identification of ocular diseases has been proven to have a great potential [75, 76], for example, key molecules and pathways affected by glaucoma pathology can be developed into a predictive diagnosis strategy [77]. Meanwhile, microbiome phenotypes can be utilized as parameters of predictive medicine, aiding in the recognition of patients’ predispositions and assessment of treatment responses [35]. The incorporation of various phenotype markers enables effective monitoring of microbiome modulation. In our case, we identified hub genes and pathways linked POAG with gut microbiota, which could be further used as biomarkers indicating POAG at an early stage from common non-invasive examination including fecal examinations.

The modification of the gut microbiota in metabolic syndrome and associated chronic diseases is among the leading tasks of microbiome research and is needed for the clinical use of probiotics [36].

Recent evidence has shown that dietary interventions have the potential to counteract systemic immunological disorders and neuroinflammation diseases by modulating and restoring gut microbiota balance [27, 78]. However, there is a lack of evidence for the implications of microbiome modification in improving metabolic health, particularly when applied in a personalized manner. In the field of developing new strategy for glaucoma in the manner of 3P medicine, probiotics also have strong and well-documented preventive potential. Thus, the gut-eye-axis hypothesis is still an emerging field of research, and further studies are needed to fully understand the mechanisms behind this relationship and to establish a causal link towards future predictive and preventive medicine.

In the end, our study has its limitations. We showed gut microbiota could be influencing the occurrence of glaucoma via affecting macrophages, but there is lack of evidence from patients with glaucoma and gut microbiota dysbiosis at the same time. Moreover, the sample number of POAG patients is relatively small (*n* = 4). As a matter of fact, the human trabecular meshwork tissue is very rare, and incomparable with other animal or in vitro cell models. Considering our results are statistically significant, we believe that the four groups of samples can still reflect the situation of POAG. Meanwhile, the condition of systemic diseases was not reported for both the 4 POAG patients and the 4 controls. Recent studies have reported in POAG patients, a significant prevalence of systemic diseases is observed, including arterial hypertension, dyslipidemia, and diabetes [7, 8, 79, 80]. Given that all three mentioned systemic diseases are known to induce inflammatory process and macrophages activation, there is a possibility of overlapping effects in the context of findings related to POAG.

In a word, as a pilot study, our observations provide expanding insight of the gut-eye-axis and possible therapeutic target for future predictive, preventive, and personalized treatment of glaucoma.

## Conclusion and expert recommendations

This study identified IL18, TLR9, NFKB1, HDAC4, FKBP2, and KITLG as hub genes in glaucoma that are gut microbiota-related as well as showed that the regulation of immune response by gut microbiota is associated with glaucoma. Those key molecules and pathway could contribute to the early prediction of POAG, thus, reduce the negative effect such as economic and clinical burden associated with unnecessary medical treatments. Our findings offered strong evidence to prove the existence of the gut-eye-axis and support the potential of systemic immune therapy for targeting preventive and personalized treatment of POAG, especially for those with gut microbiota dysbiosis [81], which could further provide expanding insight in-line with the paradigm shift from a reactive treatment to a 3PM.

## Supplementary Information

Below is the link to the electronic supplementary material.

Supplementary file1 (DOCX 95 KB)

## Data Availability

Datasets of POAG patients (GSE138125) were downloaded from the Gene Expression Omnibus (GEO) database (http://www.ncbi.nlm.nih.gov/geo/). Datasets of gut microbiota were obtained from gutMGene (http://bio-annotation.cn/gutmgene/home.dhtml).

## Code availability

All software applications used are included in this article.
