# Supplementary figures and images for: The correlation between primary open-angle glaucoma (POAG) and gut microbiota: a pilot study towards predictive, preventive, and personalized medicine
Source: EPMA J. 2023 Aug 11;14(3):539–52. doi: 10.1007/s13167-023-00336-2 (PMC10439875; doi:10.1007/s13167-023-00336-2)

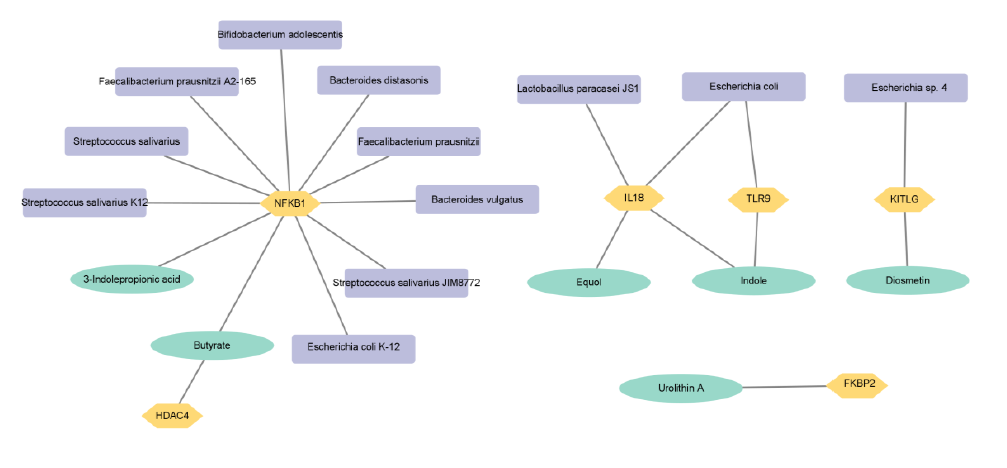


Supplement Figure 1. Relationship network of hub genes, gut microbiota and metabolites.

Supplement: Supplementary file 1 — Supplementary file1 (DOCX 95 KB) [file 13167_2023_336_MOESM1_ESM.docx]
